# Learning Delayed Influences of Biological Systems

**DOI:** 10.3389/fbioe.2014.00081

**Published:** 2015-01-16

**Authors:** Tony Ribeiro, Morgan Magnin, Katsumi Inoue, Chiaki Sakama

**Affiliations:** ^1^The Graduate University for Advanced Studies (Sokendai), Tokyo, Japan; ^2^National Institute of Informatics, Tokyo, Japan; ^3^Institut de Recherche en Communications et Cybernétique de Nantes (IRCCyN), Nantes, France; ^4^Department of Computer and Communication Sciences, Wakayama University, Wakayama, Japan

**Keywords:** Boolean network, gene regulatory networks, delayed influences, time delay, logic programming, machine learning, state transitions

## Abstract

Boolean networks are widely used model to represent gene interactions and global dynamical behavior of gene regulatory networks. To understand the memory effect involved in some interactions between biological components, it is necessary to include delayed influences in the model. In this paper, we present a logical method to learn such models from sequences of gene expression data. This method analyzes each sequence one by one to iteratively construct a Boolean network that captures the dynamics of these observations. To illustrate the merits of this approach, we apply it to learning real data from bioinformatic literature. Using data from the yeast cell cycle, we give experimental results and show the scalability of the method. We show empirically that using this method we can handle millions of observations and successfully capture delayed influences of Boolean networks.

## 1 Introduction

### 1.1 Immediate versus delayed influences

Thanks to the development of recent high-throughput measurement technologies such as DNA microarrays, biologists succeed in obtaining a large amount of gene expression profiles. It then becomes crucial to be able to connect the data and build a predictive model of the gene network. The analysis of biological networks often requires agreeing on an appropriate mathematical or computational model to represent the biological system. Because of the complexity of the system, models usually assume that the modification of one node results in an immediate activation (or inhibition) of its targeted nodes. But this hypothesis is generally unfair: some influences may take some time to operate, thus modifying the behavior of the model. These delayed influences can play a major role in various biological systems of crucial importance, like the mammalian circadian clock [as illustrated by Comet et al. (2012)] or the DNA damage repair [as shown by Abou-Jaoudé et al. (2009)]. We especially need to capture the memory of the system, i.e., keep track of the previous steps.

### 1.2 Modeling delayed influences into Boolean networks

Delayed influences have been integrated in each of the most well-known formalisms to model gene regulatory networks. Thanks to their structure, which allows to model both sequentiality and parallelism, Petri nets were able to model complex regulation mechanisms (Chaouiya, 2007). Recent works even considered not only discrete but continuous delays (Siebert and Bockmayr, 2006; Comet et al., 2010) in some hybrid automata paradigms. However, such approaches, because of their complexity, fail to deal with large systems and biological data about quantitative time delays are generally scarce. That is why we chose to focus, in this paper, on Boolean networks, which have proven to be a simple, yet powerful, framework to model and analyze the dynamics of gene regulatory networks. The classical dynamics of Boolean networks is based on the central assumption that a homogeneous transmission delay is involved among all components of the network. This means the modification of one node results in an immediate activation (or inhibition) of its targeted nodes [as studied, e.g., by Akutsu et al. (2003)] for the sake of simplicity. This is quite unrealistic in the sense that, in a real biochemical system, the evolution happens at various time scales.

**Figure 1:**
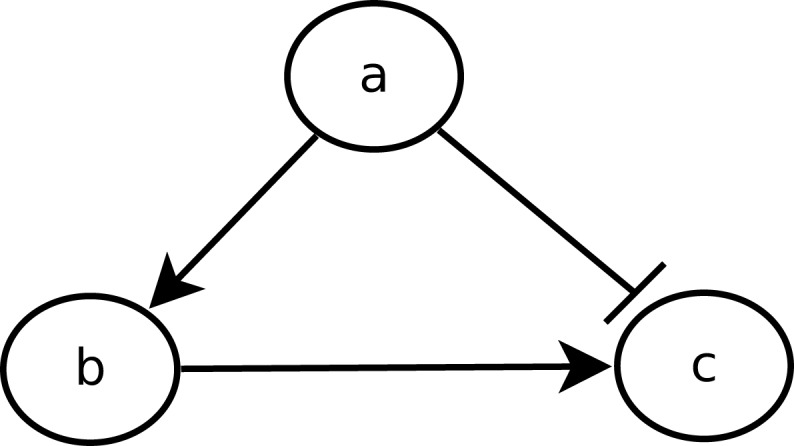
**Example of a feedforward loop**.

The urge to incorporate delays into the model is perfectly illustrated by the feedforward loop scheme. The feedforward loop is a network pattern that appears in many cycling processes, e.g., *Escherichia coli* and *Yeast Saccharomyces cerevisiae* [as considered by Koh et al. (2009)]. Biologists like Mangan and Alon (2003) assume these loops play a major role in the acceleration of the response time of transcriptional networks. It consists of the following elements (see Figure 1): 3 genes, let us say *a*, *b*, and *c*, with *a* regulating *b*, *b* regulating *c*, and a direct regulation from *a* to *c*. Depending on the nature of the regulations (activations or inhibitions; Figure 1 arbitrarily considers an inhibition from *a* to *c*) and their delays, the concurrence between the direct regulation from *a* onto *c* and the indirect one through *b* can lead to a drastic different behavior. To analyze feedforward loops, the information about the respective delays of the regulations at stake are crucial. A small change in the delays understanding may lead to a complete different behavior. The thin analysis of the dynamical behavior of such pattern requires to enrich classical discrete models with delays.

To address this issue, different approaches have been designed. The most well-known one is due to Silvescu and Honavar (2001). To understand precisely the dynamics of some biological processes (like cell development) while considering the memory of the system, the authors take their inspiration from the works about Markov models. They introduced an extension of Boolean Networks from a Markov(1) to Markov(k) model, where *k* is the number of time steps during which a gene can influence another gene. This extension is called temporal Boolean networks, abridged as *TBN*(*n*, *m*, *k*), with *n* the number of genes and the expression of each gene at time *t* + 1 being controlled by a Boolean function of the expression levels of at most *m* genes at times in {*t*, *t* − 1, …, *t* − (*k* + 1)}. They even extend the formalism to multi-valued discrete networks, calling it temporal discrete networks, *TDN*(*n*, *m*, *k*, *D*), where each gene can be expressed at levels 0, 1, …, *D* − 1. Their main results, however, focus on *TBN*(*n*, *m*, *k*): they design a decision tree learning algorithm that infers a temporal Boolean network from time series data. They also give some bounds on the size of the necessary data to infer temporal Boolean networks. They illustrate their results through artificially generated networks, claiming that the main limit of their method is the lack of real data on large datasets.

Other authors addressed the idea of modeling delayed and indirect influences. We can cite the work of Chueh and Lu (2012), who extended the Boolean network formalism with delays. To model that the induction of a Boolean function may not activate immediately the targeted gene, they replace the classical deterministic relation between the Boolean function and the targeted gene by two relationships: (i) the prerequisite function: it represents the fact that the on-status of the target gene at time *t* + 1 requires that the Boolean function at time *t* is on. (ii) The similarity function: the Boolean function and the target gene are said to be similar if the status of the Boolean function and the status of the target gene are in the same expression. In other words, this means that the classical Boolean operators are encoded in the prerequisite function, while the similarity function allows to model precedence (under the form of delays) between concurrent updates.

Another approach to model indirect influences is given in the 6th chapter of the dissertation by Ghanbarnejad (2011). The author recalls that the time passing between production of a regulating molecule and its binding to a target site depends both on the molecule and its target site. That is why he decides to study the dynamics in such a way that:

$$x_{i}\left(t\right)=f_{i}\left(x_{1}\left(t-\tau_{i1}\right),\;x_{2}\left(t-\tau_{i2}\right),\;\ldots ,\;x_{N}\left(t-\tau_{\mathrm{iN}}\right)\right)$$

with *x_i_* the value of gene *i*, *t* the current time step, τ*_ij_* the delay for the interaction between a source node *j* and a target node *i*. For the purpose of his research, the author draws the delay τ*_ij_* as a random integer from a flat distribution on $\left\{1,2\bar{\tau }-1\right\}$ for each pair of nodes i and j, the average delay $\bar{\tau }$ being a tunable parameter. That is introduced as Boolean networks with distributed delays. The semantics here is synchronous, thus very similar to what we aim at.

As these works derive of the seminal formalism proposed by Silvescu and Honavar (2001), we will consider *TBN*(*n*, *m*, *k*) in this paper and discuss a new learning algorithm.

### 1.3 Learning Boolean networks with delayed influences

Various approaches have been recently designed to tackle the reverse engineering of gene regulatory networks from expression data. This has led to the emergence of the so-called executable biology, whose goal is to provide formal methods to automatically synthesize models from experiments (Koksal et al., 2013). Most of them are only static. But there has been a growing interest for inference algorithms that incorporate temporal aspects. Koh et al. (2009) recently studied the relevance of these various algorithms. Liu et al. (2004) proposed to infer time-delayed gene regulatory networks through Bayesian networks. Lopes and Bontempi (2013) showed that the inference algorithms that include temporal features perform better than static ones. The main issue is then to be able to infer the appropriate temporal delays between the influences at stake. As this is a hard problem, Zhang (2008) claimed that the key issue when analyzing time series data consists in segmenting time series data in different successive phases. Their contribution then focuses on solving this segmentation problem and shows the merits of their approaches on various case studies.

All these approaches consider gene expression data as input and infer the associated regulations. One common problem of discrete approaches taking expression data as input lies in the determination of a relevant threshold to define the inactive and active states of gene expression. To position this hypothesis in the context of existing approaches to process raw biological data, let us cite the works of some authors, like Soinov et al. (2003), who proposed an alternative methodology that considers not a concentration level, but the way the concentration is changed in the presence/absence of one regulator. The other major modeling problem depends on the quality of the expression data. In other words, noisy data may lead to errors in the inference process. For example, when a gene is expressed at a low level, a low signal-to-noise ratio would result in an inaccurate measurement of the behavior of the gene.

The pre-processing of the data is really critical to the relevance of the inferred relations between components. In this paper, we assume our input data has already been pre-processed and resulted in a reliable set of state-transitions information.

Aside from these intrinsic modeling issues, the existing learning approaches share some computational limitations:

- Because of the complexity of the problem, the size of the inferred model is limited: the inferred gene regulatory network has to be composed of less than 15 components and the memory effect cannot take into account more than *k* = 15 steps.
- Many approaches fail when the network involves cyclic interactions.

### 1.4 Our contribution

In this paper, we focus on the logical approach to learn gene regulatory networks with delays from an existing knowledge that is expressed through a set of state transitions. As mentioned in the previous subsection, we assume there has been a pre-processing of the time series data.

In previous works, we exhibited the links between logic programs and Boolean networks on the one hand (Inoue, 2011; Inoue and Sakama, 2012), designed an algorithm that is able to learn Markov(1) state transition systems on the other hand (Inoue et al., 2014). While existing works did not allow to capture the delayed influences between components, this paper designs an algorithm that takes multiple sequences of state transitions as input and builds a logic program that capture the delayed dynamics of a Markov(*k*) system.

This can be seen as an extension of previous results in the following sense: in Inoue (2011) and Inoue and Sakama (2012), Markov(1) state transition systems are represented with logic programs, in which the state of the world is represented by a Herbrand interpretation and the dynamics that rule the environment changes are represented by a logic program *P*. The rules in *P* specify the next state of the world as a Herbrand interpretation through the *immediate consequence operator* (also called the *T_P_ operator*) [as introduced by Van Emden and Kowalski (1976) and Apt et al. (1988)]. With such a background, Inoue et al. (2014) have recently proposed a framework to learn logic programs from traces of interpretation transitions (LFIT). We extended this body of research: while the previous algorithm dealt only with 1-step transitions (i.e., we assume the state of the system at time *t* depends only of its state at time *t* − 1), we propose here an approach that is able to consider *k*-step transitions (sequence of at most *k* state transitions). This means that we are now able to capture delayed influences in the *inductive logic programing* methodology.

### 1.5 Outline of the paper

The paper is organized as follows: Section 2 reviews the logical background of this work, and summarizes the main ideas behind the existing LF1T algorithm in order to make its extension to Markov(*k*) models (i.e., with delayed influences) in Section 3 be more understandable. In Section 4, we apply our methodology to some case studies and highlight its scalability. Finally, we discuss these results and further works in Section 5.

## 2 Background

### 2.1 Boolean network

**Figure 2:**
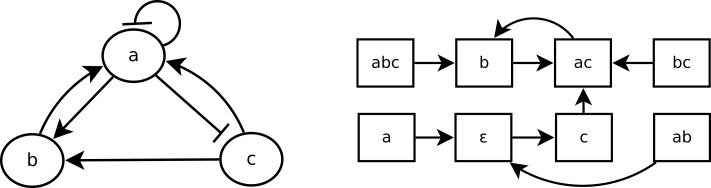
**A Boolean network *B*_1_ (left) and its state transition diagram (right)**.

A Boolean network is a simple discrete representation widely used in bioinformatics (Kauffman, 1969; Lähdesmäki et al., 2003; Klamt et al., 2006). A *Boolean network* (Kauffman, 1969) is a pair (*N*, *F*) with *N* = {*n*_1_, …, *n_k_*}, a finite set of nodes (or variables), and *F* = {*f*_1_, …, *f_k_*}, a corresponding set of Boolean functions $f_{i}:B^{n}\to B$, with B  = {0, 1}. *n_t_*(*t*) represents the value of *n_i_* at time step *t*, and equals either 1 (expressed) or 0 (not expressed). A vector (or *state*) $s(t)=(n_{1}(t),\ldots ,n_{k}(t)$ is the expression of the nodes of *N* at time step *t*. There are 2*^k^* possible distinct states for each time step. The state of a node *n_i_* at the next time step *t* + 1 is determined by $n_{i}(t+1)=f_{i}(n_{i_{1}}(t),\;\ldots ,\;n_{i_{p}}(t))$ with $n_{i_{1}},\;\ldots ,\;n_{i_{p}}$ the nodes directly influencing *n_i_*, called *regulation nodes* of *n_i_*. A Boolean network can be represented by its *interaction graph* (see Figure 2 left), but its precise regulation relations can only be represented by the Boolean function (see Example 1). For each Boolean network, there is the *state transition diagram* (see Figure 2 right), which represents the transitions between *n_i_*(*t*) and *n_i_*(*t* + 1). In the case of a gene regulatory network, nodes represent genes and Boolean functions represent their relations.

**Example 1**: Figure 2 shows the interaction graph and the state transitions diagram of a Boolean network *B*_1_ composed of the three following variables: {*a*, *b*, *c*}. The Boolean functions of *B*_1_ are *f_a_*, *f_b_*, and *f_c_*, which are, respectively, the following Boolean functions of *a*, *b*, and *c*:

$$f_{a}=\neg a\wedge \left(b\vee c\right),\;f_{b}=a\wedge c,\;f_{c}=\neg a$$

Let us consider that the Boolean network *B*_1_, whose graph is depicted in Figure 2, is a gene regulatory network so that *a*, *b*, and *c* are genes. According to the interaction graph of *B*_1_: *a* is not only an *activator* of *b* and an *inhibitor* of *c* but also its own inhibitor. The gene *b* is an activator of *a*, and the gene *c* is activator of both *a* and *b*. According to the Boolean functions of *B*_1_ in Example 1, to activate *a*, either *b* or *c* has to be present but if *a* is present, it will prevent its own expression at the next step (*f_a_*). The activation of *b* requires both *a* and *c* to be expressed at the same time step; if one of them is not expressed at time step *t* then *b* will not be expressed at *t* + 1 (*f_b_*). The presence of *a* is enough to prevent the expression of *c*, so that if *a* is expressed at time step *t* then *c* will not be expressed at *t* + 1 (*f_c_*).

It is straightforward to generate the state transition diagram from the Boolean functions. Learning from interpretation transition (LFIT) tackles the inverse problem: infer the Boolean function from state transitions. In a Boolean network, the value of nodes can be updated synchronously or asynchronously. In a *synchronous Boolean network*, all nodes are updated at the same time. The successive sequence of states during an execution, called *trajectory* of a Boolean network, is deterministic in a synchronous Boolean network. In an *asynchronous Boolean network*, a node may not be updated at a time, so that its state transitions can be non-deterministic. In this paper, we deal only with synchronous ones.

### 2.2 Logic programing

In this subsection, we recall some preliminaries of logic programing. We consider a propositional language L that is built from a finite set of propositional constants *p*, *q*, *r*, … and the logical connectives ¬, ∧ and ←. A propositional constant *p* is also called an *atom* and ¬*p* is negation of *p*. *p* and ¬*p* are called *literals*.

In this paper, we consider a *logic program* (simply called a program) as a set of *rules* of the form

(1) $$p\leftarrow p_{1}\wedge \cdots \wedge p_{m}\wedge \neg p_{m+1}\wedge \cdots \wedge \neg p_{n}$$

where *p* and *p_i_*’s are atoms (*n* ≥ *m* ≥ 1). For any rule *R* of the form (1), the atom *p* is called the *head* of *R* and is denoted as *h*(*R*), and the conjunction to the right of ← is called the *body* of *R*. We represent the set of literals in the body of *R* of the form (1) as $b\left(R\right)=\left\{p_{1},\ldots ,p_{m},\neg p_{m+1},\ldots ,\neg p_{n}\right\}$, and the atoms appearing in the body of *R* positively and negatively as *b*^+^(*R*) = {*p*_1, …,_ *p_m_*} and *b*^-^(*R*) = {*p_m_*_+1, …,_ *p_n_*}, respectively. A rule *R* of the form (1) is interpreted as follows: *h*(*R*) is true if all elements of *b*^+^(*R*) are true and none of the elements of *b*^−^(*R*) is true. When *b*^+^(*R*) = *b*^−^(*R*) = ∅, the rule is called a *fact rule*. The rule (1) is a *Horn clause* iff m = n.

**Definition 1** (Herbrand base): the Herbrand Base of a program *P*, denoted by *ℬ*, is the set of all atoms in the language of *P*.

**Definition 2** (Interpretation): let *ℬ* be the Herbrand Base of a logic program *P*. An *interpretation* is a subset of *ℬ*. If an interpretation is the empty set, it is denoted by ϵ.

**Definition 3** (Model): an interpretation *I* is a *model* of a program *P* if *b*^+^(*R*) ⊆*I* and $b^{-}\left(R\right)\cap I=\emptyset$ imply *h*(*R*) ∈ *I* for every rule R in P.

For a logic program *P* and an interpretation *I*, the *immediate consequence operator* (or *T_P_ operator*) (Apt et al., 1988) is the mapping T_P_ : 2^*ℬ*^→2^*ℬ*^:

(2) $$T_{P}\left(I\right)=\left\{h\left(R\right)|R\in P,\;b^{+}\left(R\right)\subseteq I,\;b^{-}\left(R\right)\cap I=\emptyset \right\}.$$

In the rest of this paper, we represent the state transitions of a logic program *P* as a set of pairs of interpretations (*I*, *T_P_*(*I*)).

**Definition 4** (Consistency): let *R* be a rule and (*I*, *J*) be a state transition. *R* is *consistent* with (*I*, *J*) iff *b*^+^(*R*) ⊆ *I* and *b*^−^(*R*) ∩ *I* = ∅ imply *h*(*R*) ∈*J*. Let *E* be a set of state transitions, *R* is *consistent* with *E* if *R* is consistent with all state transitions of *E*. A logic program *P* is *consistent* with *E* if all rules of *P* are *consistent* with *E*.

**Definition 5** (Subsumption): let *R*_1_ and *R*_2_ be two rules. If *h*(*R*_1_) = *h*(*R*_2_) and *b*(*R*_1_) ⊆ *b*(*R*_2_) then *R*_1_ *subsumes R*_2_. Let *P* be a logic program and *R* be a rule. If there exists a rule *R*′ ∈*P* that subsumes *R* then *P subsumes R*.

We say that a rule *R*_1_ is *more general* than another rule *R*_2_ if *R*_1_ subsumes *R*_2_.

**Example 2**: let *R*_1_ and *R*_2_ be the two following rules: *R*_1_ = (*a* ←*b*), *R*_2_ = (*a* ←*a*∧*b*), *R*_1_ subsumes *R*_2_ because (*b*(*R*_1_) = {*b*}) ⊂ (*b*(*R*_2_) = {*a*, *b*}). When *R*_1_ appears in a logic program *P*, *R*_2_ is useless for *P*, because whenever *R*_2_ can be applied, *R*_1_ can be applied.

### 2.3 Learning from interpretation transitions

*LF1T* (Inoue et al., 2014) is an *any time algorithm* that takes a set of one-step state transitions *E* as input. These one-step state transitions can be considered as positive examples. From these transitions, the algorithm learns a logic program *P* that represents the dynamics of *E*. To perform this learning process, we can iteratively consider one-step transitions. When the state transition diagram in Figure 2 is given as input to *LF1T*, it can learn the Boolean network *B*_1_.

In *LF1T*, the set of all atoms *ℬ* is assumed to be finite. In the input *E*, a state transition is represented by a pair of interpretations (subset of *ℬ*). The output of *LF1T* is a logic program that realizes all state transitions of *E*.

#### Learning from 1-step transitions (LF1T)

**Input:** *E* ⊆2*^*ℬ*^* × 2*^*ℬ*^*: (positive) examples/observations.
**Output:** A logic program *P* such that *J* = *T_P_*(*I*) holds for any (*I*, *J*) ∈*E*.

To build a logic program with *LF1T*, we use a bottom-up method that generates hypotheses by *specialization* from the most general rules that are fact rules, until the logic program is consistent with all input state transitions. Learning by specialization ensures to output the most general valid hypothesis (Ribeiro and Inoue, 2014). Here, the notion of prime implicant is used to define minimality of logic programs. We consider that the logic program learned by **LF1T** is minimal if the body of each rule constitutes a prime implicant to infer the head.

**Definition 6** (Prime implicant condition): let *R* be a rule and *E* be a set of state transitions such that *R* is consistent with *E*. *b*(*R*) is a prime implicant condition of *h*(*R*) for *E* if there is no rule *R*′ such that *b*(*R*′) ⊂ *b*(*R*) and *R*′ is consistent with E. Let *P* be a logic program such that *P*∪{*R*} ≡*P*: all models of *P*∪{*R*} are models of *P* and vice versa. *b*(*R*) is a prime implicant condition of *h*(*R*) for *P* if there is no rule *R*′ such that *P*∪{*R*′} ≡*P* and *b*(*R*′) ⊂*b*(*R*).

For the sake of simplicity, according to Definition 3, we will call *R* a minimal rule of *E* (resp. *P*) if *b*(*R*) is a *prime implicant condition* of *h*(*R*) for *E* (resp. *P*). For any atom *p*, the most general minimal rule is the rule with an empty body (*p* ←) that states that the variable is always true in the next state, i.e., a fact.

**Example 3**: Let *R*_1_, *R*_2,_ and *R*_3_ be three rules and *E* be the set of state transitions of Figure 2 as follows: *R*_1_ = *a* ←*a*∧*b*∧*c*, *R*_2_ = *a* ←*a*∧*b*, *R*_3_ = *a* ←*b*. The only rule more general than *R*_3_ is *R*′ = *a*, but *R*′ is not consistent with (*a*, ϵ) ∈ *E* so that *R*_3_ is a minimal rule for *E*. Since *R*_3_ subsumes both *R*_1_ and *R*_2_, they are not minimal rules of *E*. Let *P* be the logic program {*a* ← *b*, *b* ← *a* ∧ *c*, *c* ← ¬*a*}. *R*_3_ is a minimal rule of *P* because *P* realizes *E* and *R*_3_ is minimal for *E*.

In *Inductive Logic Programing*, refinement operators usually add a set of literals to the body of a rule to make it more specific (Muggleton and De Raedt, 1994). It is a way to revise the current knowledge to make it consistent with new information. Similarly, in this algorithm, when a rule is not consistent with the observations, we refine it by adding literals into its body.

**Definition 7** (Minimal specialization): let *R*_1_ and *R*_2_ be two rules such that *h*(*R*_1_) = *h*(*R*_2_) and *R*_1_ subsumes *R*_2_. The minimal specialization *ms*(*R*_1_, *R*_2_) of *R*_1_ over *R*_2_ is

$$\mathrm{ms}\left(R_{1},R_{2}\right)=\left\{h\left(R1\right)\leftarrow b\left(R1\right)\wedge \neg l_{i}|l_{i}\in b\left(R2\right)\setminus b\left(R1\right)\right\}$$

Minimal specialization can be used on a rule *R* to avoid the subsumption of another rule with a minimal reduction of the generality of *R*. By extension, minimal specialization can be used on the rules of a logic program *P* to avoid the subsumption of a rule with a minimal reduction of the generality of *P*. Let *P* be a logic program, *R* be a rule and *S* be the set of all rules of *P* that subsume *R*. The minimal specialization *ms*(*P*, *R*) of *P* by *R* is as follows:

$$ms(P,R)=(P\backslash S)\cup (\underset{R_{P}\in S}{\cup }ms(R_{P},R))$$

**LF1T** starts with an initial logic program P={p← ∣ p∈B}. Then **LF1T** iteratively analyzes each transition (*I*, *J*) ∈ *E*. For each variable *A* that **does not appear** in *J*, **LF1T** infers an **anti-** **rule** $R_{A}^{I}$:

$$R_{A}^{I}=A\leftarrow \underset{B_{i}\in I}{\wedge }B_{i}\wedge \underset{C_{j}\in (B\backslash I)}{\wedge }\neg C_{j}$$

A rule of *P* that subsumes such an anti-rule is not consistent with the transitions of *E* and must be revised. The idea is to use minimal specialization to make *P* consistent with the new transitions (*I*, *J*) by avoiding the subsumption of all anti-rules $R_{A}^{I}$ inferred from (*I*, *J*). After minimal specialization, *P* becomes consistent with the new transition while remaining consistent with all previously analyzed transitions. When all transitions of *E* have been analyzed, **LF1T** outputs the rules of the system that realize *E*.

## 3 Learning Markov(*k*) Systems

In order to learn Markov(*k*) when *k* > 1, we need to extend the **LF1T**. To achieve this goal, we introduce the **LFkT** algorithm. While only its essence was presented in Ribeiro et al. (2014), we formalize, in this section, the corresponding ideas and, in the next section, illustrate its merits on biological case studies taken from the literature.

### 3.1 Formalization

**Definition 8** (Timed Herbrand base): let *P* be a logic program. Let *ℬ* be the Herbrand base of *P* and *k* be a natural number. The timed Herbrand base of *P* (with period k) denoted by *ℬ_k_*, is as follows:

$$B_{k}=\overset{k}{\underset{i=1}{\cup }}\left\{v_{t-i}|v\in B\right\}$$

where *t* is a constant term, which represents the current time step.

According to Definition 1, given a propositional atom *v*, *v_j_* is a new propositional atom for each *j* = *t* − *i* (0 ≤ *i* ≤ *k*). A Markov(*k*) system can then be interpreted as a logic program as follows.

**Definition 9** (Markov(*k*) system): let *P* be a logic program, *ℬ* be the Herbrand base of *P* and *ℬ*_k_ be the timed Herbrand base of *P* with period *k*. A *Markov(k) system S* with respect to *P* is a logic program where for all rules *R* ∈ *S*, *h*(*R*) ∈ *ℬ* and all atoms appearing in *b*(*R*) belong to *ℬ*_k_.

In a Markov(*k*) system *S*, the atoms that appear in the body of the rules represent the value of the atoms that appear in the head, but at previous time steps. In a context of modeling gene regulatory networks, these latter atoms represent the concentration of the interacting genes. This concentration is abstracted as a Boolean value modeling the fact that it is lower or greater than a threshold.

**Example 4**: Let *R*_1_ and *R*_2_ be two rules, R_1_ = a ← b_t−1_ ∧ b_t−2_, R_2_ = b ← a_t−2_ ∧¬b_t−2_. The logic program *S* = {*R*_1_, *R*_2_} is a Markov(2) system, i.e., the state of the system depends on the two previous states. The value of *a* is true at time step *t* only if *b* was true at *t* − 1 and *t* − 2. The value of *b* is true at time step *t* only if *a* was true at *t* − 2 and *b* was false at *t* − 2. The atoms that appear in the head of the rules of *S* is {*a*, *b*}. *ℬ*_1_ represents these atoms from time step *t* − 1: *ℬ*_1_ = {*a_t_*_−1_, *b_t_*_−1_} and *ℬ*_2_ represents these atoms from time step *t* − 2: *ℬ*_2_ = {*a_t_*_−1_, *b_t_*_−1_, *a_t_*_−2_, *b_t_*_−2_}.

In the following definitions, we refer to ℕ as the set of all natural numbers.

**Definition 10** (Trace of execution): let *B* be the atoms that appear in the head of the rules of a Markov(*k*) system *S*. A *trace of execution T* is a finite sequence of states of *S*: *T* = (*S*_0_, …, *S_n_*), *n* ≥ 1, ∀*i* ∈*N*, *i* ≤ *n*, *S_i_* ∈ 2*^B^*. For all *j* ∈ ℕ, we define:

$$\mathrm{prev}\;\left(i,j,T\right)=\left\{\begin{array}{cc} \emptyset & \mathrm{if}\;i=0\;\mathrm{or}\;j=0,\\ \left(S_{i-j-1},\ldots ,S_{i-1}\right) & \mathrm{if}\;j+1\leq i\\ \left(S_{0},\ldots ,S_{i-1}\right) & \mathrm{otherwise}. \end{array}\right.$$

We also define *prev*(*i*, *T*) = *prev*(*i*, *n*, *T*) and *next*(*i*′, *T*) = *S*_*i*′+1_, *i*′∈ ℕ, *i*′ < *n*.

We denote by |*T*| the size of the trace that is the number of elements of the sequence. A sub-trace of size *m* of a trace of execution *T* is a sub-sequence of consecutive states of *T* of size *m*, where *m* ∈ ℕ, 1 < *m* ≤ |*T*|. In the following, we will also denote *T* = (*S*_0_, …, *S_n_*) as *T* = *S*_0_ →… →*S_n_*.

**Definition 11** (Consistent traces): let *T* = (*S*_0_, …, *S_n_*) and $T\prime =\left(S_{0}^{\prime },\;\ldots ,\;S_{m}^{\prime }\right)$ be two traces of execution. *T* and *T*′ are *k*-*consistent*, with *k* ∈ ℕ, iff ∀*i*, *j* ∈ ℕ, *i* < *n*, *j* < *m*, *S_i_* = *S_j_* and *next*(*i*, *T*)≠*next*(*j*, *T*′) imply *prev*(*i*, *k*, *T*)≠*prev*(*j*, *k*, *T*′). *T* and *T*′ are said *consistent* iff they are max(*n*, *m*) consistent.

**Figure 3:**
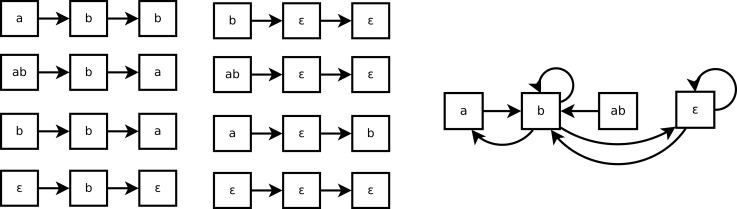
**Eight traces of executions of the system of Example 4 (left) and the corresponding state transitions diagram (right)**.

As shown in Figure 3, a Markov(*k*) system may seem non-deterministic when it is represented by a state transition diagram (right part of the figure). That is because such state transition diagram only represents 1-step transitions. In this example, the transition from the state *b* is not Markov(1): the next state can be either *a*, *b* or ∈. But it can be Markov(2), because all traces of size 2 of Figure 3 are consistent.

**Definition 12** (*k*-step interpretation transitions): let *P* be a logic program, *ℬ* be the Herbrand base of *P* and *ℬ*_k_ be the timed Herbrand base of *P* with period *k*. Let *S* be a Markov(*k*′) system w.r.t *P*, *k*′ ≥ *k*. A *k*-*step interpretation transition* is a pair of interpretations (*I*, *J*) where *J* ⊆ *ℬ*_k_ and *J* ⊆ *ℬ*.

**Example 5**: The trace *ab* →*b* →*a* can be interpreted in the following three ways:

- (*a_t_*_−2_*b_t_*_−2_*b_t_*_−1_, *a*): the 2-step interpretation transition that corresponds to the full trace *ab* →*b* →*a*.
- (*a_t_*_−1_*b_t_*_−1_, *b*): the 1-step interpretation transition corresponding to the sub-trace *ab* →*b*.
- (*b_t_*_−1_, *a*): the 1-step interpretation transition that corresponds to the sub-trace *b* →*a*.

**Definition 13** (Extended consistency): let *R* be a rule and (*I*, *J*) be a *k*-step interpretation transition. *R* is *consistent* with (*I*, *J*) iff *b*^+^(*R*) ⊆*I* and *b*^−^(*R*)∩*I* = ∅ imply *h*(*R*) ∈*J*. Let *T* be a sequence of state transitions, *R* is *consistent* with *T* if it is consistent with every *k*-step interpretation transitions of *T*. Let *O* be a set of sequences of state transitions, *R* is *consistent* with *O* if *R* is consistent with all *T*′ ∈*O*.

### 3.2 Algorithm

Here, we briefly summarize the essence of LFkT. Because of the lack of space, the details of the algorithm, its pseudo-code, and the proofs of correctness are given as Supplementary Material. We refer to the pseudo-code of the appendix as follows: (algo.N l.x-y) for Algorithm N, line x to y. **LFkT** is an algorithm that can learn the dynamics of a Markov(*k*) system from its traces of executions. **LFkT** takes a set of traces of executions *O* as input, where each trace is a sequence of state transitions. If all traces are consistent, the algorithm outputs a logic program *P* that realizes all transitions of *O*. The learned influences can be at most *k*-step relations, where *k* is the size of the longest trace of *O*. The main idea is to extract *n*-step interpretation transitions, 1 ≤ *n* ≤ *k*, from the traces of executions of the system. Transforming the traces into pairs of interpretations allows us to use minimal specialization (Ribeiro and Inoue, 2014) to iteratively learn the dynamics of the system.

**LFkT**:

- **Input**: A set of traces of execution *E* of a Markov(*k*) system *S*.
- Step 1: Initialize *k* logic programs with facts rules.
- Step 2: Convert the input traces of executions into interpretation transitions.
- Step 3: Revise iteratively the logic programs by all interpretation transitions using minimal specialization.
- Step 4: Merge all logic programs into one.
- **Output**: The rules of *S*, which generated *E*.

The idea of the algorithm is to start with the most general rules (algo.1 l.6-10) and use specialization to make them consistent with the input observations (algo.2). The algorithm analyzes each interpretation transition one by one and revises the learned rules when they are not consistent (algo.1 l.13-23). In the following, we will call an *n*-step rule any rule from the logic program learned from *n*-step transitions.

After analyzing all interpretation transitions, the programs that have been learned are merged into a unique logic program (algo.1 l.24-29). This operation ensures that the rules outputted are consistent with all observations. It can be checked by comparing each rule with other logic programs. If an *n*-step rule *R* is more general than an *n*′-step rules *R*′, *n*′ < *n*, then *R* is not consistent with the observations from which *R*′ has been learned. To avoid this case, we can remove *n*-step rules that have no variable of the form *v_t_* _− _*_n_*. Indeed, if such rules are consistent with the observations, then they should also have been learned from (*n* − 1)-step rules. Finally, **LFkT** outputs a logic program that realizes all consistent traces of execution of *O*.

## 4 Evaluation and Biological Case Study

In the previous subsections, we have illustrated step by step how the **LFkT** algorithm is able to learn Markov(*k*) systems. To illustrate the merits of our work, we now apply this approach to the analysis of the yeast cell cycle dataset from Spellman et al. (1998) and Cho et al. (1998), which have been previously analyzed in Li et al. (2006). In this paper, Li et al. tackle the inference of gene regulatory networks from temporal gene expression data. The originality of their work lies in the fact they consider delayed correlations between genes. The methodology can capture gene regulations that are delayed of *k* time units. The limits of the approach is that the authors only consider pairwise overlaps of expression levels shifted in time relative to each other. Another limit of the approach is that it is not able to make a distinction between a causal gene–gene regulation and the scenarios where two genes, A and B, are being co-regulated by a third gene C: do we have A that regulates B that regulates C, or is it a co-operation between A and B that regulates C?

Here, starting from a set of different traces coming from the yeast cell cycle system, we have performed various experiments where we have tuned the number of traces that have been considered on the one hand, the value of *k* (i.e., the number of time steps representing the memory of the system) on the other hand.

**Figure 4:**
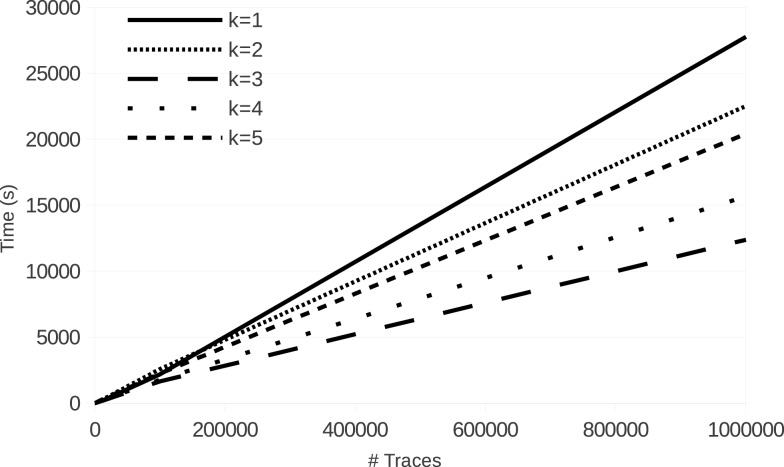
**LFkT run time varying the input size (number of traces)**.

Figure 4 shows the evolution of run time of learning with **LFkT** on the five Boolean networks of the yeast cell cycle proposed by Li et al. (2006). These fives programs are, respectively, Markov(1) to Markov(5). In these experiments, for each Boolean network, the number of variables is 16 and the length of traces in input is five states. The five Boolean networks have been implemented as a logic program using Answer Set Programing (Brewka et al., 2011). The source code of these programs is given as Supplementary Material. Traces of executions of these programs have been computed using the answer set solver clasp (Gebser et al., 2012). All experiments are run with a C++ implementation of **LFkT** on a processor Intel Xeon (X5650, 2.67GHz) with 12 GB of RAM. The main purpose of these experiments is to assess the efficiency of our approach, i.e., how many traces **LFkT** can handle for a given *k*. Complete output of LFkT for these experiments is accessible as textfile at http://tony.research.free.fr/paper/Frontier/output.zip.

In the first table of Figure 4, the evolution of run time from 10 to 1,000,000 traces (which is arbitrary chosen as upper bound of the scalability of the experiments) shows that, in practice, learning with **LFkT** is linear in the number of traces when the number of variables is fixed. Results show that the algorithm can handle more than one million of traces in less than 10 h. Since each trace is a sequence of five state transitions, when learning the Markov(5) system, each trace can be decomposed into 15 interpretation transitions (one 5-step, two 4-step, three 3-step, four 2-step, and five 1-step). Learning the Markov(5) program from one million traces of executions of size five requires the processing of 15 million of interpretation transitions. Learning the Markov(4) to Markov(1) programs requires to process, respectively, 14 million, 12 million, 9 million, and 5 million of interpretation transitions. Intuitively one could expect that learning the Markov(2) system to take significantly more time than learning the Markov(1) system. But each program is different, i.e., the Markov(2) program is not an extension of the Markov(1) program with 2-step rules. That is why run time is not always larger for a larger *k*: learning time also depends on the rules that are learned. In this experiment, the best run time is obtained with the Markov(3) program. We cannot say that the rules of this program are simpler than the others, but they are simpler to learn for the algorithm. In the second table, we observe that the number of rules learned for the Markov(3) program is significantly smaller than for the others. It means that the algorithm needs to compare less rules for each traces analysis, which can explain the speed up.

In this benchmark, in order to be faithful to the biological experiments presented by Li et al. (2006), we considered *k* = 5 as a maximum. But our algorithm succeeds in processing larger memory effects. On some random dummy examples (accessible at the above mentioned URL), we were able to learn Markov(7) systems with the following performances: we can learn 10 traces in 2.8 s, 100 traces in 27 s, 1,000 traces in 249 s, 10,000 traces in 3,621 s, 100,000 traces in 39,973 s, and 1,000,000 traces in 441,270 s. Even if the computation time increases, it should be kept in mind that our method is designed to allow successive refinements of a model about its memory effect. These results show that such an approach is tractable even with a large number of input traces.

## 5 Conclusion and Future Work

### 5.1 Summary of the contribution

To understand the memory effect involved in some interactions between biological components, it is necessary to include delayed influences in the model. In this paper, we proposed a logical method to learn such models from state transition systems. We designed an approach to learn Boolean networks with delayed influences. We have given a step by step explanation of this methodology, and illustrated its merits on a biological benchmark coming from a real-life case study.

### 5.2 Further works

Further works aim at adapting the approach developed in the paper to the kind of data produced by biologists. This requires connecting through various biological databases in order to extract real time series data, and subsequently explore and use them to learn gene regulatory networks. On account of the noise inherent to biological data, the ability to either perform an efficient discretization of the data or to include the notion of noise inside the modeling framework is fundamental. We will thus have to discuss the discretization procedure and the robustness of our modeling against noisy data and compare it to existing approaches, like the Bayesian ones (Barker et al., 2011).

Regarding the model, we consider extending the methodology to asynchronous semantics. Garg et al. (2008) addressed the differences and complementarity of synchronous and asynchronous semantics to model regulatory networks and identify attractors. The authors focus on attractors, which are central to gene regulation. Previous studies about attractors with synchronous semantics [by Melkman et al. (2010) and Akutsu et al. (2011)] and asynchronous semantics [by Bernot et al. (2004) and Fauré et al. (2006)] showed that different updating rules result in different attractors. The benefits of the synchronous model are to be computationally tractable, while classical state space exploration algorithms fail on asynchronous ones. Yet, the synchronous modeling relies on one quite heavy assumption: all genes can make a transition simultaneously and need an equivalent amount of time to change their expression level. Even if this is not realistic from a biological point of view, it is usually sufficient as the exact kinetics and order of transformations are generally unknown. The asynchronous semantics, however, helps to capture more realistic behaviors. That is why we plan to extend our approach to asynchronous semantics.

Finally, we will also address multi-valued networks that may be useful to capture behaviors that cannot be summarized through a pure Boolean framework.

## Author Contributions

Tony Ribeiro: formalization of the problem; design, implementation, description, and pseudo-code of the algorithm; design, implementation, run, and discussion of experiments. Morgan Magnin: state of the art, introduction, biological background, case study, and conclusion. Katsumi Inoue: supervision of the work; formalization of the logic programing and learning from interpretation transition approach background. Chiaki Sakama: formalization of the logic programing and learning from interpretation transition approach background.

## Conflict of Interest Statement

The authors declare that the research was conducted in the absence of any commercial or financial relationships that could be construed as a potential conflict of interest.

## Supplementary Material

The Supplementary Material for this article can be found online at http://www.frontiersin.org/Journal/10.3389/fbioe.2014.00081/abstract
