# Supplementary material for: Learning Delayed Influences of Biological Systems
Source: Front Bioeng Biotechnol. 2015 Jan 16;2:81. doi: 10.3389/fbioe.2014.00081 (PMC4296389; doi:10.3389/fbioe.2014.00081)
Supplement: Supplementary file 1 [file DataSheet_1.PDF]

Frontiers in Bioengineering

Research Article
12 December 2014

Supplementary Material: Learning Delayed Influences of Biological Systems

Tony Ribeiro [1,*], Morgan Magnin [2,3], Katsumi Inoue [2,1] and Chiaki Sakama[4]

[1] *The Graduate University for Advanced Studies (Sokendai), 2-1-2 Hitotsubashi, Chiyoda-ku, Tokyo 101-8430, Japan*
[2] *National Institute of Informatics, 2-1-2 Hitotsubashi, Chiyoda-ku, Tokyo 101-8430, Japan*
[3] *Institut de Recherche en Communications et Cybernétique de Nantes (IRCCyN), École Centrale de Nantes, 1 rue de la Noë 44321 Nantes, France*
[4] *Department of Computer and Communication Sciences, Wakayama University, Sakaedani, Wakayama 640-8510, Japan*

Correspondence*:
Tony Ribeiro
National Institute of Informatics, 2-1-2 Hitotsubashi, Chiyoda-ku, Tokyo 101-8430, Japan, tony_ribeiro@nii.ac.jp

This supplementary material provides the details of the **LFkT** algorithm of (**Ribeiro et al.**, 2014). The first section provides the pseudo code of **LFkT** together with its detailed explanations. The proof for correctness of the algorithm is also provided. Finally, we give the ASP source code of the program learned in the experiments.

1 THE LFKT ALGORITHM

LFkT is an extension of the **LF1T** algorithm (**Inoue et al.**, 2014; **Ribeiro and Inoue**, 2014) that can learn a Markov(k) system. **LFkT** takes a set of traces of executions O as input, each trace is a sequence of state transitions. If all traces are consistent, the algorithm outputs a logic program P that realizes all transitions of O. The learned influences can be at most k-step relations, where k is the size of the longest trace of O. Algorithm 1 shows the pseudo-code of **LFkT** and Algorithm 2 shows how input interpretation is done in practice.

Step 1: The algorithm starts with k logic programs $P_0^{\mathcal{B}}$. The idea is to learn rules independently for each possible k-step relation: 1-step rules, 2-step rules, . . . , k-step rules. The rules learned from 1-step interpretations will go into the 1-step program, the rules learned from 2-step interpretations will go into the 2-step program and so on. These different programs are merged at the end to constitute a logic program that realizes all consistent traces of O.

Step 2: In order to use minimal specialization, we need to convert the input traces of execution into interpretation transitions. This conversion is done by the function **interprete**, whose pseudo code is given in Algorithm 2. It extracts all k-step interpretations from each trace $T \in O$. It can be done by extracting and converting all sub-traces of T into corresponding interpretations. The function outputs them as a vector of set of interpretation transitions E, where each set E_i corresponds to interpretation of sub-traces of size i.

Algorithm 1 **LFkT**(O) : Learn the most general rules that explain E

INPUT: O a set of sequences of state transition (traces of executions)
OUTPUT: The logic program that realizes the transitions of O.

P' a vector of set of rules
E a vector of set of pairs of interpretations (I, J)
$max_k :=$ the size of the longest trace of O
// 1) Initialize P' with max_k, the logic program that contains the most general rules
for each k from 1 to max_k **do**
  $P'_k := \emptyset$
  for each $A \in \mathcal{B}$ **do**
    $P'_k := P_k \cup \{A.\}$
  $P' := P' \cup P'_k$
// 2) Extract interpretation from trace of execution
$E :=$ **interprete**(O)

// 3) Specify P' by the interpretation of the trace of execution
for each k from 1 to max_k **do**
  $E_k :=$ the kth set of interpretation of E
  while $E_k \neq \emptyset$ **do**
    Pick $(I, J) \in E_k$; $E_k := E_k \setminus \{(I, J)\}$
    for each $A \in \mathcal{B}$ **do**
      if $A \notin J$ **then**
        $R^I_A := A \leftarrow \bigwedge_{B_i \in I} B_i \wedge \bigwedge_{C_j \in (\mathcal{B} \setminus I)} \neg C_j$
        $P'_k :=$ the kth set of rule of P'
        $P'_k :=$ **Specialize**(P'_k,R^I_A)
  end while

// 4) Merge the programs into a unique logic program
$P := \emptyset$
for each k from 1 to max_k **do**
  $P'_k :=$ the kth set of rule of P'
  Remove from P'_k all rules that do not contain any literal of the form v_{t-k}
  $P := P \cup P'_k$
return P

Step 3: The algorithm iteratively learns from each set of pairs of interpretations $E_i \in E$. Now it only needs to apply the **LF1T** method of (**Ribeiro and Inoue**, 2014) on each set E_i by analyzing each pair of interpretations $(I, J) \in E_i$. For each variable A that **does not appear** in J, it infers an **anti-rule** $R^I_A := A \leftarrow \bigwedge_{B_i \in I} B_i \wedge \bigwedge_{C_j \in (\mathcal{B}_i \setminus I)} \neg C_j$, where $\mathcal{B}_i$ is the i-step atoms of E_i, i.e. all atoms that can appear in a rule of E_i. Then, minimal specialization is used to make the corresponding logic program P'_i consistent with R^I_A. Algorithm 2 of (**Ribeiro and Inoue**, 2014) shows the pseudo code of this operation (also given as appendix). In the function **specialize**, it first extracts all rules $R_P \in P$ that subsumes R^I_A.It generates the minimal specialization of each R_P by generating a rule for each literal in R^I_A. Each rule contains all literals of R_P plus the opposite of a literal in R^I_A so that R^I_A is not subsumed by that rule. Then **specialize** adds in P all the generated rules that are not subsumed by P, so that P becomes consistent with the transition (I, J).When all transitions have been analyzed, **LF1T** outputs P that has become the logic program that realizes E.

Algorithm 2 interprete(O) : Extract interpretations transition from traces

INPUT: O a set of sequences of state transitions (traces of executions)
OUTPUT: E a vector of set of pairs of interpretations (I, J)

$E := \emptyset$
// Extract interpretations
for each sequence $T \in O$ **do**
  for each k from $|T|$ to 1 **do**
    for each sub-trace T' of size k in T **do**
      $s_k :=$ the kth state of T
      $I := \emptyset$
      for each state $s_{k'}$ before s_k in T' **do**
        $t := k - k'$
        for each atom $a \in s_{k'}$ **do**
          $I := I \cup \{a_t\}$
        $E_k :=$ the kth set of interpretations of E
        $E_k := E_k \cup \{(I, s_k)\}$
return E

Algorithm 3 specialize(P,R) : specialize the logic program P to not subsume the rule R

INPUT: a logic program P and a rule R
OUTPUT: the minimal specific specialization of the rule of P by R.

$conflicts$: a set of rules
$conflicts := \emptyset$
// Search rules that need to be specialized
for each rule $R_P \in P$ **do**
  if R_P is conflicting with R **then**
    $conflicts := conflicts \cup R_P$
    $P := P \setminus R_P$
// Revise the rules by minimal specialization
for each rule $R_c \in conflicts$ **do**
  for each literal $l \in b(R)$ **do**
    if $l \notin b(R_c)$ and $\bar{l} \notin b(R_c)$ **then**
      $R'_c := (h(R_c) \leftarrow (b(R_c) \cup \bar{l}))$
      if P does not subsume R'_c **then**
        $P := P \setminus$ all rules subsumed by R'_c
        $P := P \cup R'_c$
return P

Step 4: After analyzing all interpretation transitions, the programs that have been learned are merged into a unique logic program. This operation ensures that the rule outputted are consistent with all observations. If a rule is not consistent with an observation, it has to be deleted. It can be checked by comparing each rule with other logic programs. If a n-step rule R is more general than a n'-step rules R', $n' < n$, then R is not consistent with the observations from which R' has been learned. To avoid this case, we just need to remove n-step rules that have no v_n variable. Finally, **LFkT** outputs a logic program that realizes all consistent traces of execution of O. If O is a set of traces of execution of a Boolean network, the logic program outputted by **LFkT** represents the Boolean functions of each variables. For each variable, it corresponds to the conditions over the k previous step to make it active at $t + 1$.

THEOREM 1 (Correctness of **LFkT**). *Let O be a set of traces of execution of a Markov(k) system S. Using O as input,* **LFkT** *outputs a logic program that realizes all consistent traces of O.*

PROOF. Let V be the vector of interpretation transitions extracted from O by **LFkT** (Algorithm 2). According to Theorem 4 of **Ribeiro and Inoue** (2014), initializing **LF1T** with $P_0^{\mathcal{B}}$, by using minimal specialization iteratively on a set of interpretation transitions E, we obtain a logic program P that realizes E. Since **LFkT** uses **LF1T** on each element of V, **LFkT** learns a vector of logic programs P' such that each logic program $p'_n \in P'$ realizes the corresponding set of interpretation transitions $v_n \in V$.

Let $p'_n \in P'$ be the logic program learn from $v_n \in V$, $n \geq 1$. p'_n is obtained by minimal specialization of $P_0^{\mathcal{B}}$ with all anti-rule of v_n (non consistent rule). According to Theorem 3 of **Ribeiro and Inoue** (2014), p'_n does not subsume any anti-rule of v_n. Then, p'_n realizes all deterministic transition of v_n, that is $\forall(I, J) \in v_n$, $\nexists(I, J')$, $J \neq J'$ and p'_n only contains prime rules.

Since v_n contains interpretation transitions that represent all sub-traces of size n of O, p'_n realizes all consistent sub-traces of size n of O. Let P_{n-1} be a logic program that realizes all consistent sub-traces of size at most $n-1$ of O. p'_n can contain a rule R such that $(\mathcal{B}_n \setminus \mathcal{B}_{n-1}) \cap b(R) = \emptyset$ (no literal of R refers to the $t-n$ states of the variables). In this case R realizes a sub-trace of size n and also some sub-traces of size at most $n-1$. If these sub-traces of size $n-1$ are consistent, then they are necessary realized by P_{n-1}. $P_{n-1} \cup \{R\}$ does not realize more consistent sub-traces of size at most $n-1$ than P_{n-1}. Let S_R be the set of rules of p'_n of the form R, then $(p'_n \setminus S_R)$ only realizes all sub-traces of size n of O. Then the logic program $P_n = P_{n-1} \cup (p'_n \setminus S_R)$ only realizes all consistent sub-trace of size at most $n-1$ of O and all sub-traces of size n of O, that is P_n realizes all consistent sub-traces of size at most n of O.

Let $p'_1 \in P'$ be the logic program learned from $v_1 \in V$, and let $P = p'_1$. Let R' be all rules of the logic program p'_n such that $(\mathcal{B}_n \setminus \mathcal{B}_{n-1}) \cap b(R') \neq \emptyset$. Iteratively adding rules R' into P, starting by the logic program p_2 until p_k, we obtain a logic program that realizes all consistent sub-traces of size at most k of O. As a result, using O as input, **LFkT** outputs a logic program that realizes all consistent traces of O. □

2 ASP SOURCE CODE OF THE MARKOV(1) TO MARKOV(5) PROGRAMS USED IN THE EXPERIMENTS

time(0..5).

% State generation
%————————

true(cln1,T) :- not false(cln1,T), time(T). false(cln1,T) :- not true(cln1,T), time(T).

true(cln2,T) :- not false(cln2,T), time(T). false(cln2,T) :- not true(cln2,T), time(T).

true(clb1,T) :- not false(clb1,T), time(T). false(clb1,T) :- not true(clb1,T), time(T).

true(clb2,T) :- not false(clb2,T), time(T). false(clb2,T) :- not true(clb2,T), time(T).

true(clb4,T) :- not false(clb4,T), time(T). false(clb4,T) :- not true(clb4,T), time(T).

true(clb6,T) :- not false(clb6,T), time(T). false(clb6,T) :- not true(clb6,T), time(T).

true(sic1,T) :- not false(sic1,T), time(T). false(sic1,T) :- not true(sic1,T), time(T).

true(sw16,T) :- not false(sw16,T), time(T). false(sw16,T) :- not true(sw16,T), time(T).

true(cdc28,T) :- not false(cdc28,T), time(T). false(cdc28,T) :- not true(cdc28,T), time(T).

true(cdc34,T) :- not false(cdc34,T), time(T). false(cdc34,T) :- not true(cdc34,T), time(T).

true(sw15,T) :- not false(sw15,T), time(T). false(sw15,T) :- not true(sw15,T), time(T).

true(sw14,T) :- not false(sw14,T), time(T). false(sw14,T) :- not true(sw14,T), time(T).

true(cdc20,T) :- not false(cdc20,T), time(T). false(cdc20,T) :- not true(cdc20,T), time(T).

true(cic1,T) :- not false(cic1,T), time(T). false(cic1,T) :- not true(cic1,T), time(T).

% One-unit time Rules
%————————

false(cln1,T) :- true(sw15,T-1), time(T).

false(cln2,T) :- true(clb1,T-1), time(T).

false(cln3,T) :- true(cdc53,T-1), time(T).

false(clb1,T) :- true(clb6,T-1), time(T).

false(clb2,T) :- true(clb6,T-1), time(T).

false(clb4,T) :- false(cln1,T-1), time(T).

true(clb6,T) :- false(sw15,T-1), time(T).

true(cic1,T) :- true(cln3,T-1), time(T).

false(sw16,T) :- false(skp1,T-1), false(cln2,T-1), time(T).

true(cdc28,T) :- false(mbp1,T-1), time(T).

true(sw15,T) :- true(clb1,T-1), time(T).

true(sw14,T) :- true(cdc28,T-1), time(T).

false(cdc20,T) :- false(clb1,T-1), time(T).

% Traces generation
%————————

#hide. #show true/2. #show false/2.

time(0..5).

% State generation
%————————

true(cln1,T) :- not false(cln1,T), time(T). false(cln1,T) :- not true(cln1,T), time(T).

true(cln2,T) :- not false(cln2,T), time(T). false(cln2,T) :- not true(cln2,T), time(T).

true(clb1,T) :- not false(clb1,T), time(T). false(clb1,T) :- not true(clb1,T), time(T).

true(clb2,T) :- not false(clb2,T), time(T). false(clb2,T) :- not true(clb2,T), time(T).

true(clb4,T) :- not false(clb4,T), time(T). false(clb4,T) :- not true(clb4,T), time(T).

true(clb6,T) :- not false(clb6,T), time(T). false(clb6,T) :- not true(clb6,T), time(T).

true(sic1,T) :- not false(sic1,T), time(T). false(sic1,T) :- not true(sic1,T), time(T).

true(sw16,T) :- not false(sw16,T), time(T). false(sw16,T) :- not true(sw16,T), time(T).

true(cdc28,T) :- not false(cdc28,T), time(T). false(cdc28,T) :- not true(cdc28,T), time(T).

true(mcm1,T) :- not false(mcm1,T), time(T). false(mcm1,T) :- not true(mcm1,T), time(T).

true(sw15,T) :- not false(sw15,T), time(T). false(sw15,T) :- not true(sw15,T), time(T).

true(sw14,T) :- not false(sw14,T), time(T). false(sw14,T) :- not true(sw14,T), time(T).

true(cdc20,T) :- not false(cdc20,T), time(T). false(cdc20,T) :- not true(cdc20,T), time(T).

true(mbp1,T) :- not false(mbp1,T), time(T). false(mbp1,T) :- not true(mbp1,T), time(T).

% Two-unit time Rules
%————————

true(cln1,T) :- false(sw15,T-1), time(T).

false(cln2,T) :- false(sic1,T-2), time(T).

false(clb1,T) :- true(clb6,T-1), time(T).

false(clb2,T) :- true(clb6,T-1), time(T).

false(clb4,T) :- false(cln1,T-1), time(T).

true(sic1,T) :- true(clb2,T-2), time(T).

false(mbp1,T) :- true(mcm1,T-2), time(T).

true(sw15,T) :- false(sic1,T-2), time(T).

false(skp1,T) :- false(sw16,T-2), time(T).

true(cdc20,T) :- true(clb1,T-2), time(T).

% Traces generation
%————————

#hide. #show true/2. #show false/2.

time(0..5).

% State generation
%————————

true(cln1,T) :- not false(cln1,T), time(T). false(cln1,T) :- not true(cln1,T), time(T).

true(cln2,T) :- not false(cln2,T), time(T). false(cln2,T) :- not true(cln2,T), time(T).

true(clb1,T) :- not false(clb1,T), time(T). false(clb1,T) :- not true(clb1,T), time(T).

true(clb2,T) :- not false(clb2,T), time(T). false(clb2,T) :- not true(clb2,T), time(T).

true(clb4,T) :- not false(clb4,T), time(T). false(clb4,T) :- not true(clb4,T), time(T).

true(clb6,T) :- not false(clb6,T), time(T). false(clb6,T) :- not true(clb6,T), time(T).

true(sic1,T) :- not false(sic1,T), time(T). false(sic1,T) :- not true(sic1,T), time(T).

true(sw16,T) :- not false(sw16,T), time(T). false(sw16,T) :- not true(sw16,T), time(T).

true(cdc28,T) :- not false(cdc28,T), time(T). false(cdc28,T) :- not true(cdc28,T), time(T).

true(skp1,T) :- not false(skp1,T), time(T). false(skp1,T) :- not true(skp1,T), time(T).

true(sw15,T) :- not false(sw15,T), time(T). false(sw15,T) :- not true(sw15,T), time(T).

true(sw14,T) :- not false(sw14,T), time(T). false(sw14,T) :- not true(sw14,T), time(T).

true(cdc20,T) :- not false(cdc20,T), time(T). false(cdc20,T) :- not true(cdc20,T), time(T).

true(mbp1,T) :- not false(mbp1,T), time(T). false(mbp1,T) :- not true(mbp1,T), time(T).

% Three-unit time Rules
%————————

false(cln2,T) :- false(sic1,T-2), time(T).

false(clb1,T) :- true(clb6,T-1), time(T).

false(clb2,T) :- true(clb6,T-1), time(T).

true(sic1,T) :- false(cln2,T-3), time(T).

true(cdc34,T) :- false(cdc34,T-3), time(T).

true(sw15,T) :- false(sic1,T-2), time(T).

true(skp1,T) :- true(mbp1,T-3), time(T).

true(sw14,T) :- true(clb2,T-3), time(T).

true(cdc20,T) :- true(clb1,T-2), time(T).

% Traces generation
%————————

#hide. #show true/2. #show false/2.

time(0..5).

% State generation
%——————————

true(cln1,T) :- not false(cln1,T), time(T). false(cln1,T) :- not true(cln1,T), time(T).

true(cln2,T) :- not false(cln2,T), time(T). false(cln2,T) :- not true(cln2,T), time(T).

true(clb1,T) :- not false(clb1,T), time(T). false(clb1,T) :- not true(clb1,T), time(T).

true(clb2,T) :- not false(clb2,T), time(T). false(clb2,T) :- not true(clb2,T), time(T).

true(clb4,T) :- not false(clb4,T), time(T). false(clb4,T) :- not true(clb4,T), time(T).

true(clb6,T) :- not false(clb6,T), time(T). false(clb6,T) :- not true(clb6,T), time(T).

true(sic1,T) :- not false(sic1,T), time(T). false(sic1,T) :- not true(sic1,T), time(T).

true(sw16,T) :- not false(sw16,T), time(T). false(sw16,T) :- not true(sw16,T), time(T).

true(cdc28,T) :- not false(cdc28,T), time(T). false(cdc28,T) :- not true(cdc28,T), time(T).

true(skp1,T) :- not false(skp1,T), time(T). false(skp1,T) :- not true(skp1,T), time(T).

true(sw15,T) :- not false(sw15,T), time(T). false(sw15,T) :- not true(sw15,T), time(T).

true(sw14,T) :- not false(sw14,T), time(T). false(sw14,T) :- not true(sw14,T), time(T).

true(cdc20,T) :- not false(cdc20,T), time(T). false(cdc20,T) :- not true(cdc20,T), time(T).

true(mbp1,T) :- not false(mbp1,T), time(T). false(mbp1,T) :- not true(mbp1,T), time(T).

% Four-unit time Rules
%——————————

true(cln1,T) :- false(sw15,T-1), time(T).

false(cln2,T) :- false(sic1,T-2), time(T).

true(clb6,T) :- true(cln3,T-4), time(T).

true(mbp1,T) :- false(skp1,T-4), time(T).

true(sw15,T) :- false(sic1,T-2), time(T).

true(sw14,T) :- true(clb2,T-3), time(T).

false(cdc20,T) :- false(sw16,T-4), time(T).

% Traces generation
%——————————

#hide. #show true/2. #show false/2.

time(0..5).

% State generation
%——————

true(cln1,T) :- not false(cln1,T), time(T). false(cln1,T) :- not true(cln1,T), time(T).

true(cln2,T) :- not false(cln2,T), time(T). false(cln2,T) :- not true(cln2,T), time(T).

true(clb1,T) :- not false(clb1,T), time(T). false(clb1,T) :- not true(clb1,T), time(T).

true(clb2,T) :- not false(clb2,T), time(T). false(clb2,T) :- not true(clb2,T), time(T).

true(clb4,T) :- not false(clb4,T), time(T). false(clb4,T) :- not true(clb4,T), time(T).

true(clb5,T) :- not false(clb5,T), time(T). false(clb5,T) :- not true(clb5,T), time(T).

true(clb6,T) :- not false(clb6,T), time(T). false(clb6,T) :- not true(clb6,T), time(T).

true(sic1,T) :- not false(sic1,T), time(T). false(sic1,T) :- not true(sic1,T), time(T).

true(sw16,T) :- not false(sw16,T), time(T). false(sw16,T) :- not true(sw16,T), time(T).

true(cdc28,T) :- not false(cdc28,T), time(T). false(cdc28,T) :- not true(cdc28,T), time(T).

true(cdc34,T) :- not false(cdc34,T), time(T). false(cdc34,T) :- not true(cdc34,T), time(T).

true(sw15,T) :- not false(sw15,T), time(T). false(sw15,T) :- not true(sw15,T), time(T).

true(sw14,T) :- not false(sw14,T), time(T). false(sw14,T) :- not true(sw14,T), time(T).

true(cdc20,T) :- not false(cdc20,T), time(T). false(cdc20,T) :- not true(cdc20,T), time(T).

% Five-unit time Rules
%————————-

true(cln1,T) :- false(sw15,T-1), time(T).

false(cln2,T) :- false(sic1,T-2), time(T).

false(clb1,T) :- false(clb5,T-5), time(T).

false(clb2,T) :- false(clb5,T-5), time(T).

true(clb4,T) :- false(clb4,T-5), time(T).

false(clb6,T) :- false(cdc20,T-5), time(T).

true(sic1,T) :- true(clb1,T-4), time(T).

true(cdc28,T) :- false(clb6,T-5), time(T).

true(cdc34,T) :- false(cdc34,T-5), time(T).

true(sw15,T) :- false(sic1,T-2), time(T).

true(sw14,T) :- false(clb5,T-5), time(T).

false(cdc20,T) :- false(sw16,T-4), time(T).

% Traces generation %——————————

#hide. #show true/2. #show false/2.

REFERENCES

Inoue, K., Ribeiro, T., and Sakama, C. (2014), Learning from interpretation transition, Machine Learning, 94, 1, 51–79

Ribeiro, T. and Inoue, K. (2014), Learning prime implicant conditions from interpretation transition, in The 24th International Conference on Inductive Logic Programming, to appear (long paper) (http://tony.research.free.fr/paper/ILP2014long)

Ribeiro, T., Magnin, M., and Inoue, K. (2014), Learning delayed influence of dynamical systems from interpretation transition, in The 24th International Conference on Inductive Logic Programming, to appear (short paper) (http://tony.research.free.fr/paper/ILP2014short)
